# Transcription of *DWARF4* Plays a Crucial Role in Auxin-Regulated Root Elongation in Addition to Brassinosteroid Homeostasis in *Arabidopsis thaliana*

**DOI:** 10.1371/journal.pone.0023851

**Published:** 2011-08-31

**Authors:** Yuya Yoshimitsu, Kiwamu Tanaka, Wataru Fukuda, Tadao Asami, Shigeo Yoshida, Ken-ichiro Hayashi, Yuji Kamiya, Yusuke Jikumaru, Tomoaki Shigeta, Yasushi Nakamura, Tomoaki Matsuo, Shigehisa Okamoto

**Affiliations:** 1 Department of Agricultural Sciences and Natural Resources, Kagoshima University, Kagoshima, Japan; 2 Division of Plant Sciences, University of Missouri, Columbia, Missouri, United States of America; 3 Department of Applied Biological Chemistry, The University of Tokyo, Bunkyo-ku, Tokyo, Japan; 4 RIKEN, Plant Science Center, Tsurumi-ku, Yokohama, Kanagawa, Japan; 5 Department of Biochemistry, Okayama University of Science, Okayama, Japan; 6 Department of Biochemical Science and Technology, Kagoshima University, Kagoshima, Japan; 7 Department of Food Sciences and Nutritional Health, Kyoto Prefectural University, Shimogamo, Sakyo-ku, Kyoto, Japan; Iwate University, Japan

## Abstract

The expression of *DWARF4* (*DWF4*), which encodes a C-22 hydroxylase, is crucial for brassinosteroid (BR) biosynthesis and for the feedback control of endogenous BR levels. To advance our knowledge of BRs, we examined the effects of different plant hormones on *DWF4* transcription in *Arabidopsis thaliana*. Semi-quantitative reverse-transcriptase PCR showed that the amount of the *DWF4* mRNA precursor either decreased or increased, similarly with its mature form, in response to an exogenously applied bioactive BR, brassinolide (BL), and a BR biosynthesis inhibitor, brassinazole (Brz), respectively. The response to these chemicals in the levels of β-glucuronidase (*GUS*) mRNA and its enzymatic activity is similar to the response of native *DWF4* mRNA in *DWF4::GUS* plants. Contrary to the effects of BL, exogenous auxin induced GUS activity, but this enhancement was suppressed by anti-auxins, such as α-(phenylethyl-2-one)-IAA and α-*tert*-butoxycarbonylaminohexyl-IAA, suggesting the involvement of SCF^TIR1^-mediated auxin signaling in auxin-induced *DWF4* transcription. Auxin-enhanced GUS activity was observed exclusively in roots; it was the most prominent in the elongation zones of both primary and lateral roots. Furthermore, auxin-induced lateral root elongation was suppressed by both Brz application and the *dwf4* mutation, and this suppression was rescued by BL, suggesting that BRs act positively on root elongation under the control of auxin. Altogether, our results indicate that *DWF4* transcription plays a novel role in the BR-auxin crosstalk associated with root elongation, in addition to its role in BR homeostasis.

## Introduction

Brassinosteroids (BRs) are a class of plant hormones with a unique polyhydroxy steroid structure. BRs regulate not only various developmental processes such as cell division, cell elongation and xylem differentiation under genetic control, but also plant architecture and metabolism in response to continuously changing environments [1]. In addition, BRs contribute to the expression of biotic and abiotic stress tolerance in a variety of plants and promote increased yields in some crop plants [2], [3], which suggests a potential for future applications in agriculture.

To date, many aspects of BR biosynthesis, metabolism and signal transduction have been revealed by both molecular genetic and biochemical research [4], [5]. Through these studies, numerous BR biosynthesis genes have been identified and characterized. Among them, the *Arabidopsis DWARF4* (*DWF4*) gene has been of interest because it encodes a C-22 hydroxylase that catalyzes multiple key regulatory steps in BR biosynthesis and is essential for the normal growth and development of plants. Severe dwarfism results from the depletion of endogenous BRs caused by null mutations in *DWF4* [6] or by the application of the BR biosynthesis inhibitor brassinazole (Brz) to wild-type plants, which directly binds and inhibits the DWF4 enzyme [7]. Furthermore, *DWF4* is critical for the maintenance of BR homeostasis because its expression changes in a rapid, signal-dependent fashion in response to endogenous BR levels [8], [9]. However, the mechanisms behind the feedback expression of the *DWF4* are still unknown.

It is generally believed that several plant hormones, rather than the individual hormones, govern each developmental process through their complex interactions at multiple levels, including biosynthesis, metabolism, transport and signaling [10]. BRs and auxin have been shown to act together on many developmental processes, including leaf expansion, stem elongation and vascular differentiation, cooperatively in some cases and antagonistically in others [11]. BRs and auxin act synergistically to enhance hypocotyl elongation [12], [13] and lateral root development [14], whereas the two hormones function antagonistically with respect to gravitropism in hypocotyls and roots [15], [16]. Moreover, DNA microarray and quantitative reverse-transcriptase PCR (qRT-PCR) analyses have indentified target genes common to both BRs and auxin, such as *GH3*, *SAUR-AC1* (*SAUR15*) and some of the *Aux/IAA* genes, which suggests crosstalk between the BR and auxin signaling pathways [13], [17]. It has also been reported that, in addition to signaling, BR and auxin effects converge at the levels of hormone transport and biosynthesis. BRs promote lateral root formation through the enhancement of acropetal auxin transport [14]; in contrast, auxin indirectly induces the *CONSTITUTIVE PHOTOMORPHOGENESIS AND DWARFISM* (*CPD*) gene, which encodes a BR biosynthesis enzyme by up-regulating the *BREVIS RADIX* (*BRX*) gene [18].

In this study, we investigated the transcriptional response of the *DWF4* gene to different plant hormones, including BR, to dissect the role of *DWF4* expression in BR homeostasis and plant growth and development. Here, we provide evidence that *DWF4* transcription is involved in auxin-regulated root growth in addition to the feedback regulation of endogenous BR levels.

## Results

### *DWF4* transcription in response to endogenous BR content

We previously revealed that the level of *DWF4* mRNA rapidly changed in response to the application of a bioactive BR, BL and a BR biosynthesis inhibitor, Brz, suggesting that *DWF4* expression plays a crucial role in BR homeostasis [9]. The involvement of transcriptional control in the BL-mediated down-regulation of *DWF4* expression has so far been studied molecularly; the corresponding *cis*-element (BRRE) and transcription factor (BZR1) have been identified [19], whereas the transcriptional control of Brz-mediated *DWF4* up-regulation has rarely been investigated, with the exception of the discovery, by Kim et al. [20], of an increase in β-glucuronidase (*GUS*) mRNA levels and GUS staining in *DWF4::GUS* transgenic plants. To confirm and verify these findings, we performed the two types of kinetic analyses described below.

**Figure 1:**
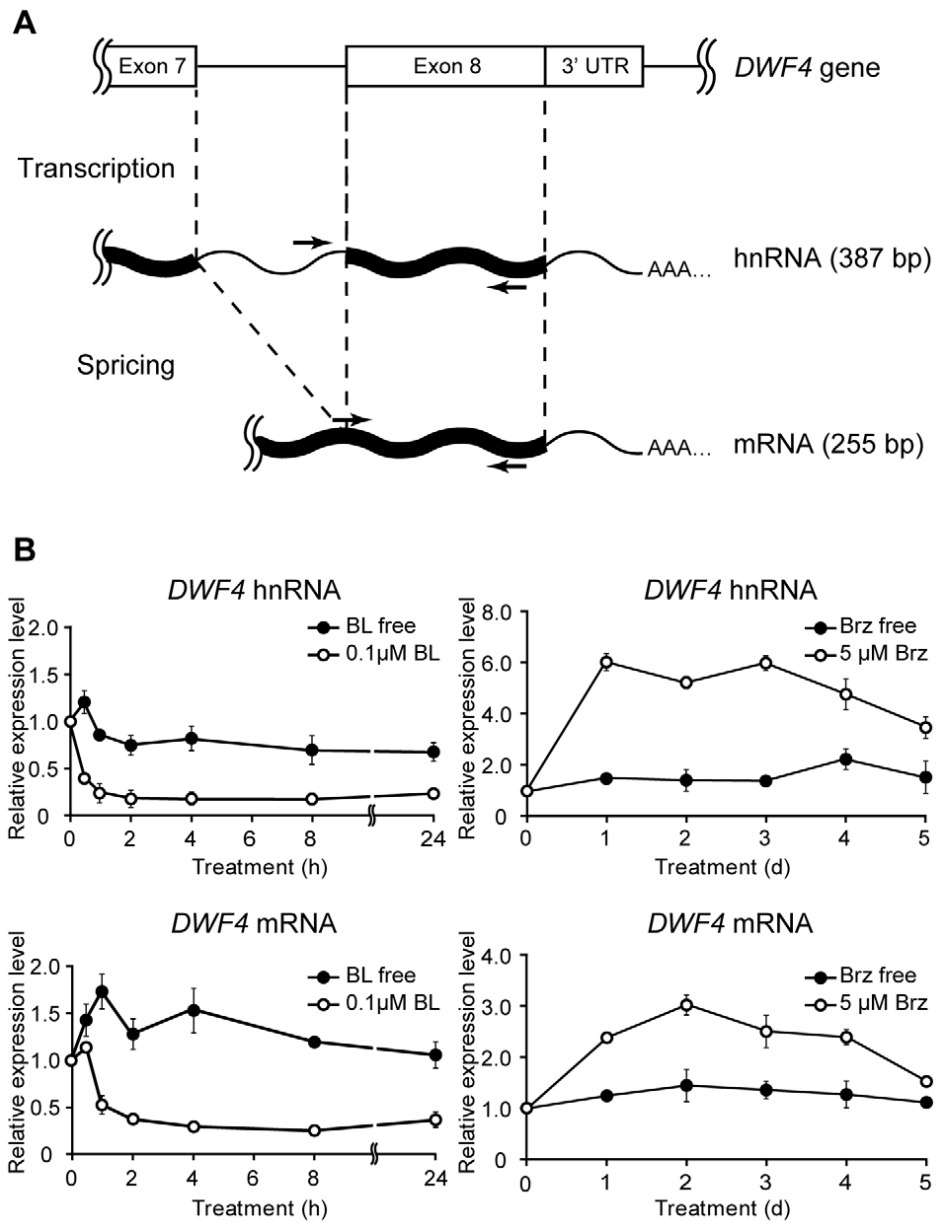
Fluctuations in *DWF4* hnRNA and *DWF4* mRNA levels dependent on BR content in wild-type *Arabidopsis*. Using semi-qRT-PCR, *DWF4* hnRNA and mRNA levels were measured to evaluate the effects of Brz and BL on *DWF4* transcription. To detect both RNA species, we designed two types of primer (arrow) sets, shown in a schematic diagram of gene expression. The lengths (bp) of the PCR products derived from both RNAs are shown in parentheses (A). Fourteen-day-old wild-type *Arabidopsis* plants were cultured in liquid MS medium containing 5 µM Brz for the indicated number of days to evaluate the effect of Brz, whereas the seedlings were incubated in the same medium containing 5 µM Brz for 2 days and then cultured in the presence of 0.1 µM BL for the indicated number of hours to evaluate the effect of BL (B). The fluorescence intensity of ethidium bromide of each PCR product band was scanned with a fluoro-image analyzer (FLA 2000; Fujifilm, Tokyo, Japan) after electrophoresis. The relative fluorescence values are shown in graphs comparing the initial period after normalization with that of *ACT2*, which was used as an internal control. Each experiment was conducted in biologically triplicate, and the means ± SE were calculated.

The amount of heterogeneous nuclear RNA (hnRNA) is generally assumed to reflect the transcription rate of the corresponding gene because hnRNA is a transient intermediate that is quickly processed to generate mRNA [21]. Therefore, we compared the levels of hnRNA and mRNA, both of which were derived from a single *DWF4* gene, in wild-type *Arabidopsis*, using semi-qRT-PCR (Figure 1). To amplify both RNA species, we designed two different forward primers; one corresponded to the sequence in the seventh intron of the *DWF4* gene used for hnRNA detection, and the other matched the sequence at the junction region between the seventh and eighth exons used for mRNA detection (Figure 1A). Upon the application of 0.1 µM BL, both the hnRNA and mRNA levels of *DWF4* rapidly decreased within 2 h and remained at minimum levels for at least 24 h (Figure 1B). In contrast, *DWF4* hnRNA significantly increased and reached a maximum level within 1 day in response to the application of 5 µM Brz, and then it gradually declined during the following 4 days, consistent with the accumulation pattern of *DWF4* mRNA.

**Figure 2:**
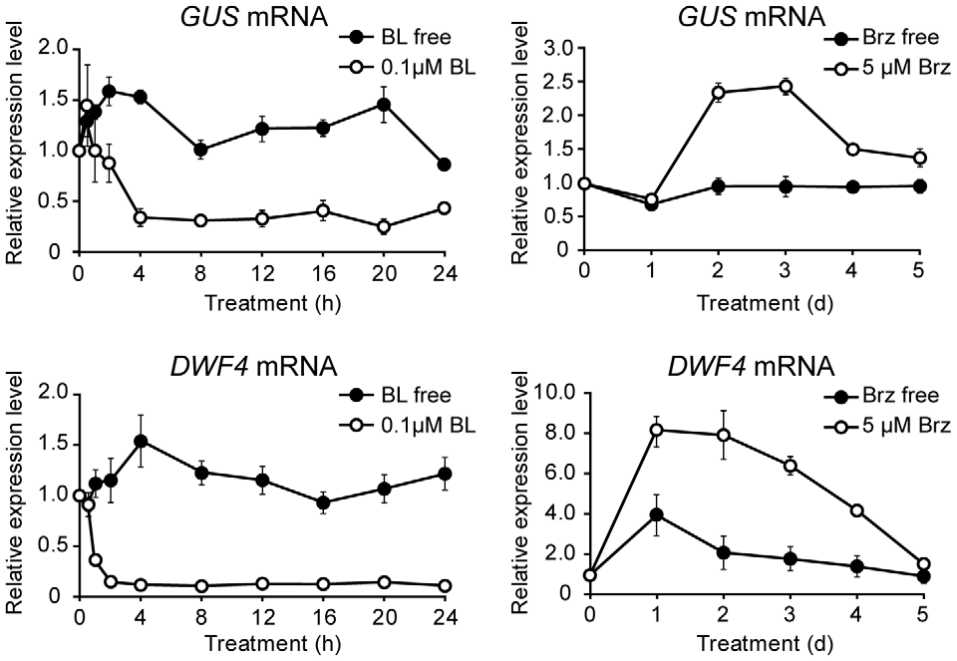
Fluctuations in *DWF4::GUS* and native *DWF4* mRNA levels dependent on BR levels in transgenic *Arabidopsis*. Semi-qRT-PCR was performed to examine the expression of *DWF4::GUS* and native *DWF4* in response to exogenously applied Brz or BL. The culture conditions were the same as those described in Figure 1. The relative fluorescence value is shown in graphs comparing the initial period after normalization with that of the *ACT2* used as an internal control. Each experiment was conducted in biologically triplicate, and the means ± SE were calculated.

Subsequently, we investigated the level of *GUS* mRNA driven by a 1.7-kb upstream sequence of *DWF4* (*DWF4::GUS*) to further confirm the above data. As shown in Figure 2, endogenous *DWF4* mRNA and *GUS* mRNA levels were altered in response to both BL and Brz in a transgenic *Arabidopsis* plant. After BL application, the mRNA levels of both *DWF4* and *GUS* decreased in the same manner within 4 h. However, the change in mRNA levels differed between *DWF4* and *GUS* in response to Brz: the *GUS* mRNA level increased from day 2 and declined at day 4, whereas the endogenous *DWF4* mRNA level increased from day 1, kept its maximum level by day 2 and then gradually decreased by day 5. In addition, the enhancement of *GUS* mRNA by Brz was less than that of *DWF4* mRNA.

**Figure 3:**
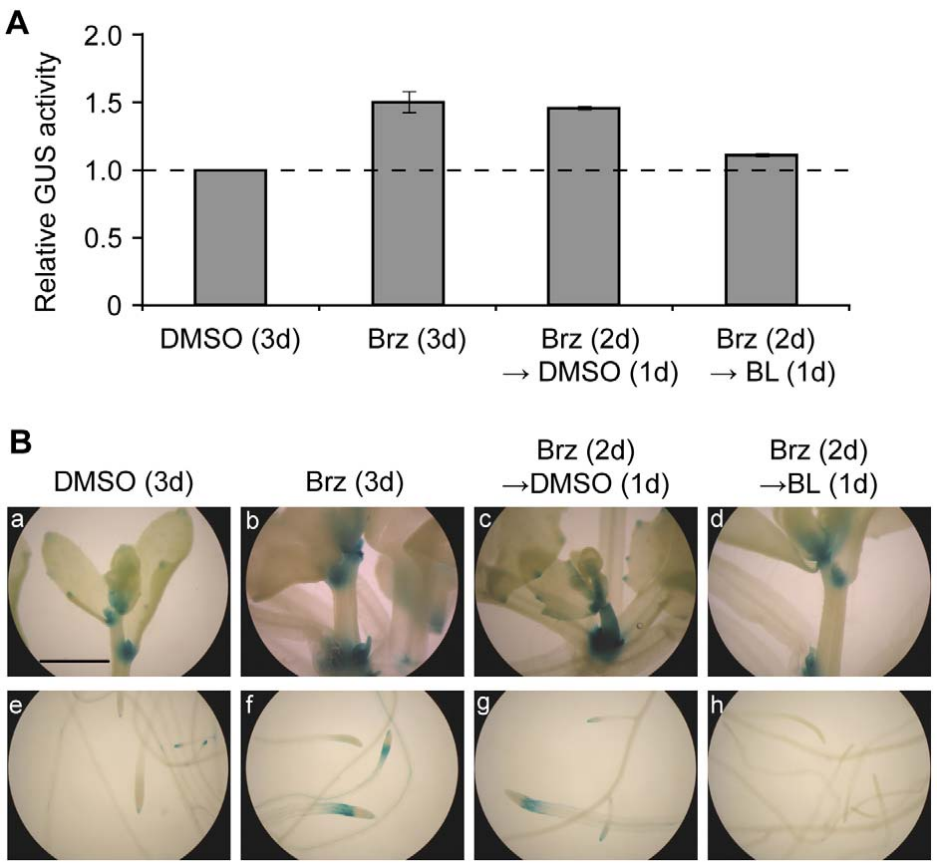
The changes in DWF4::GUS activity and staining dependent on BR content. GUS activity was analyzed biochemically (A) and histochemically (B) to examine the effects of Brz and BL on *DWF4* transcription. Brz (5 µM) was applied to 14-day-old *DWF4::GUS* transgenic seedlings and then the seedlings were cultured for 3 days in liquid MS medium to evaluate the effect of Brz, whereas Brz (for 2 days) and BL (0.1 µM; for 1 day) were sequentially applied to evaluate the effect of BL. The graphs show representative results among three independent experiments. GUS assays in each experiment were conducted in triplicate, and the means ± SD were calculated. GUS activity is shown as a value relative to that of the untreated control. The photographs represent the shoots (a–d) and roots (e–h) of transgenic *Arabidopsis*. The magnification is the same in all photographs, and a bar indicates 1.0 mm (a).

Furthermore, we biochemically and histochemically examined the GUS activity derived from the *DWF4::GUS* transgene to determine the BR dependency and tissue specificity of *DWF4* transcription. Consistent with the fluctuation in the *DWF4:GUS* mRNA level (Figure 2), GUS activity in a whole seedling was enhanced by 5 µM Brz for 3 days and was reduced to the untreated control level by 0.1 µM BL treatment for 1 day, following 5 µM Brz treatment for 2 days (Figure 3A). In the absence of these chemicals, GUS staining was observed in the shoot apices, the elongation zones of the primary roots and the lateral root primordia and tips (Figure 3B). Upon the application of 5 µM Brz for 3 days, GUS staining was strongly intensified in both the shoot apices and elongation zones of the primary roots. In contrast, GUS staining was decreased to the level of the untreated control by the serial application of 5 µM Brz (for 2 days) and 0.1 µM BL (for 1 day). No additional tissues were stained with the GUS activity after either Brz or BL treatment, suggesting that feedback transcription of *DWF4* is mostly restricted to both the shoot apices and elongation zones of the primary roots.

### Auxin-regulated *DWF4* transcription

**Figure 4:**
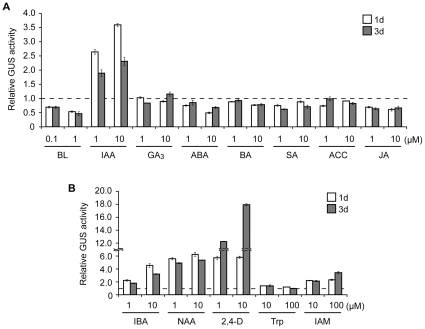
The induction of DWF4::GUS activity by bioactive auxins. Fourteen-day-old *DWF4::GUS* transgenic plants were incubated in liquid MS medium containing one of either eight different plant hormones (A), three bioactive auxins or their precursors (B) for 1 or 3 days. GUS activity was measured biochemically. In the graphs, the GUS activity (the mean ± SD) of a representative result among three biological replicates is shown as a value relative to that of the untreated control. BL, brassinolide; IAA, indole-3-acetic acid; GA_3_, gibberellic acid; ABA, abscisic acid; BA, benzyl adenine; SA, salicylic acid; ACC, 1-aminocyclopropane-1-carboxylic acid; JA, jasmonic acid; IBA, indole-3-butylic acid; NAA, α-naphtylacetic acid; 2,4-D, 2,4-dichlorophenoxyacetic acid; Trp, l-tryptophan; IAM, indole-3-acetamide.

To determine whether *DWF4* transcription is controlled by plant hormones other than BRs to further elucidate DWF4 functions. Thus, we biochemically measured GUS activity in *DWF4::GUS* plants treated with one of eight plant hormones, including BRs (Figure 4A). Indole-3-acetic acid (IAA) significantly increased GUS activity in the transgenic plants, and 10 µM IAA was more effective than 1 µM for both of the incubation periods (1 and 3 days). This enhancement of *DWF4* transcription by IAA was further confirmed by dose-dependence and time-course studies (Figure S1). In contrast, the remaining six hormones barely increased GUS activity, and only methyl jasmonate (MeJA) decreased GUS activity in 1 and 3 days at both concentrations (1 and 10 µM).

Because IAA exclusively enhanced GUS activity, we concentrated on auxins in the subsequent experiments. To confirm the auxin inducibility of *DWF4* transcription, *DWF4::GUS* seedlings were treated with various bioactive auxins or auxin precursors (Figure 4B). The elevation of GUS activity by three bioactive auxins, indole-3-butyric acid (IBA), α-naphthylacetic acid (NAA) and 2,4-dichlorophenoxyacetic acid (2,4-D), was similar to the elevation by IAA. The synthetic auxins (NAA and 2,4-D) were more effective than the natural ones (IAA and IBA). In contrast, GUS activity was barely increased by l-tryptophan (Trp), and it was slightly induced by indole-3-acetamide (IAM) at concentrations 10 to 100 times higher than the effective concentrations of bioactive auxins.

**Figure 5:**
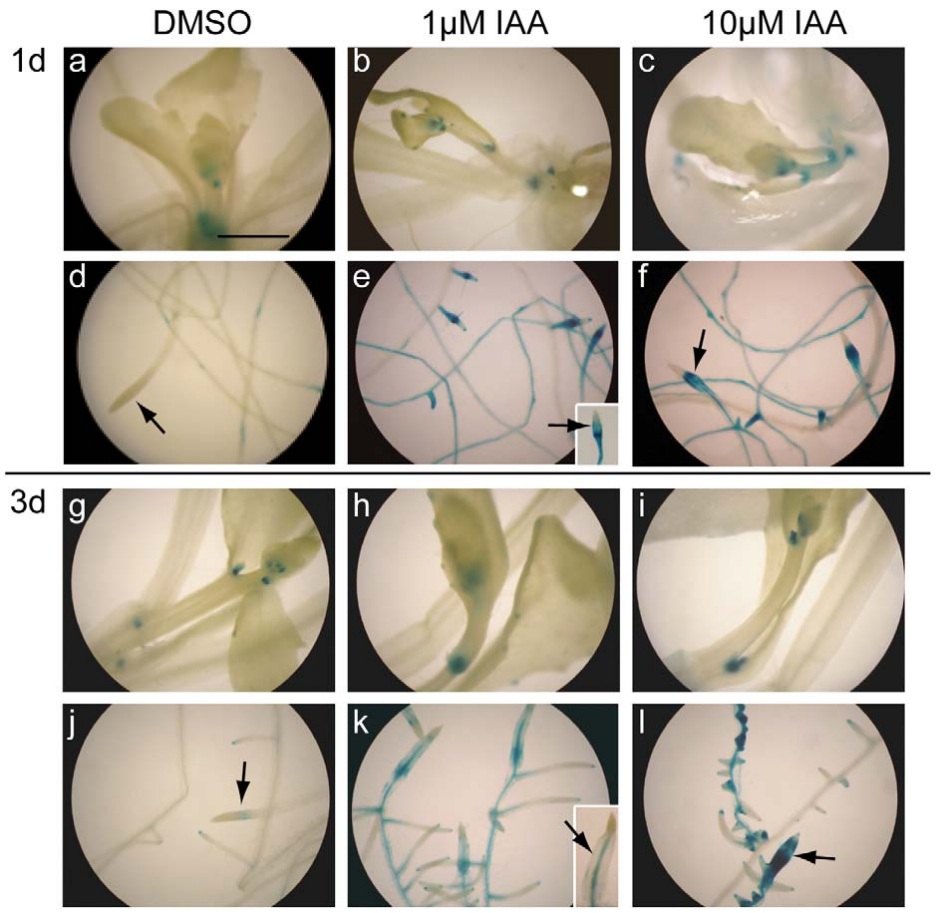
The root-specific induction of DWF4::GUS activity by IAA. *DWF4::GUS* transgenic plants (14 days old) were incubated in liquid MS medium containing various concentrations of IAA for the indicated periods. Then, GUS activity was examined histochemically. The photographs represent the shoots (a–c, g–i) and roots (d–f, j–l) of transgenic *Arabidopsis*. The arrows indicate primary root tips (d–f, j–l). Primary root tips are shown in insets when they are absent in the main flames of photographs (e and k). The magnification is the same in all photographs, and the bar indicates 1.0 mm (a).

We then performed histochemical assays, and as shown in Figure 5, IAA enhanced GUS activity in the roots; the elongation zones of both the primary and lateral roots and the lateral root primordia were most strongly stained. However, IAA did not enhance GUS staining in the shoot system, including the apices (Figure 5), whereas Brz did (Figure 3B). Furthermore, IAA induced lateral root initiation and caused swelling of the primary root tips, as previously reported [22]; this was especially evident 3 days after IAA addition (Figure 5, j–l).

**Figure 6:**
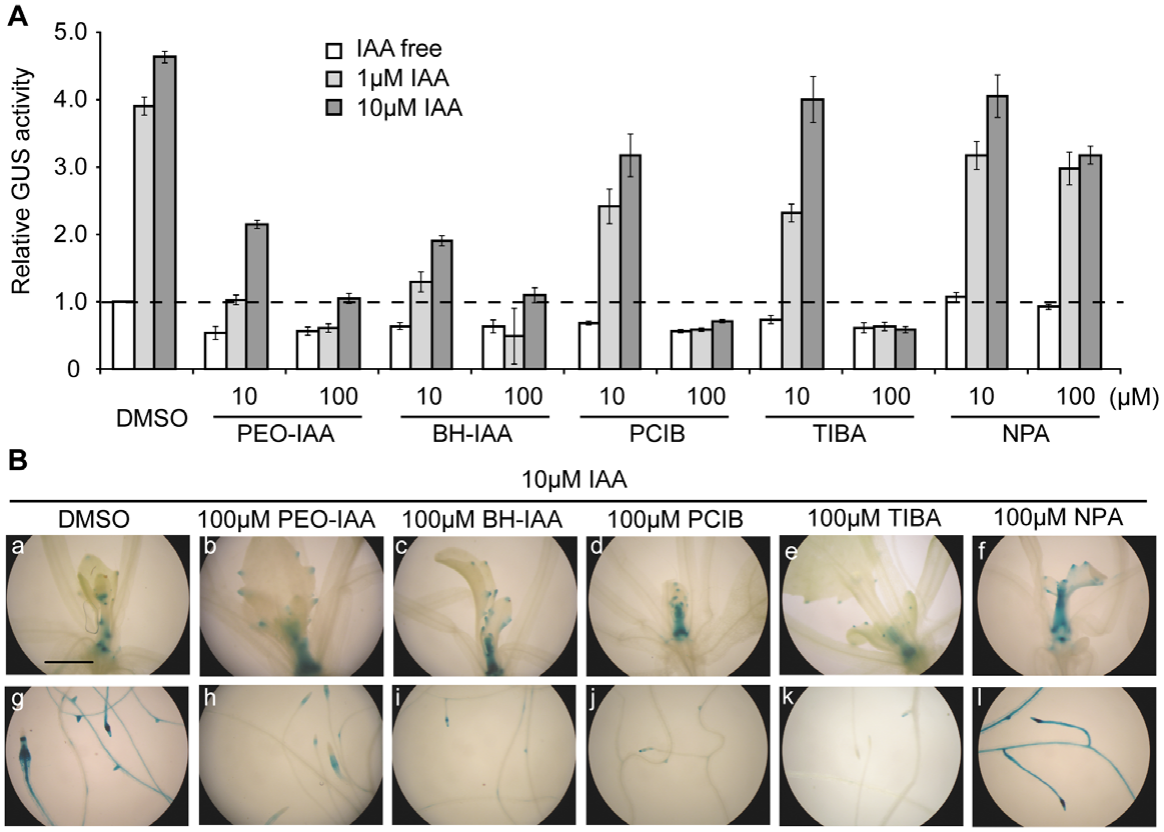
The inhibitory effects of three anti-auxins and the PAT inhibitor TIBA on IAA-induced DWF4::GUS activity. Fourteen-day-old *DWF4::GUS* transgenic seedlings were cultured for 1 day in liquid MS medium containing IAA and one of either three anti-auxins (PEO-IAA, BH-IAA or PCIB) or two PAT inhibitors (TIBA or NPA) at the indicated doses. GUS activity was analyzed biochemically (A) and histochemically (B). In the graphs, the GUS activity (the mean ± SD) of a representative result among three independent measurements is shown as a value relative to that of the untreated control. The photographs represent the shoots (a–f) and roots (g–l) of transgenic *Arabidopsis*. The magnification is same in all the photographs, and the bar indicates 1.0 mm (a). PCIB, 2-(p-chlorophenoxy)-2-methylpropionic acid; PEO-IAA, α-(phenylethyl-2-one)-IAA; BH-IAA, α-*tert*-butoxycarbonylaminohexyl-IAA; TIBA, 2,3,5-triiodobenzoic acid; NPA, N-1-naphthylphthalamic acid.

To investigate whether *DWF4* transcription is governed by auxin signaling or by polar auxin transport (PAT), IAA was applied to *DWF4::GUS* seedlings together with one of the following inhibitors: 2-(p-chlorophenoxy)-2-methylpropionic acid (PCIB), α-(phenylethyl-2-one)-IAA (PEO-IAA) or α-*tert*-butoxycarbonylaminohexyl-IAA (BH-IAA) as anti-auxins and 2,3,5-triiodobenzoic acid (TIBA) or N-1-naphthylphthalamic acid (NPA) as PAT inhibitors. As shown in Figure 6A, IAA-induced GUS activity was strongly suppressed by both PEO-IAA and BH-IAA, which are direct inhibitors of the SCF^TIR1^ auxin receptor [22], [23], at both 10 and 100 µM. In contrast, IAA-induced GUS activity was slightly inhibited by 10 µM PCIB and completely suppressed by 100 µM PCIB, which suggests different inhibitory effects among the three anti-auxins. Among the PAT inhibitors, 100 µM TIBA strongly inhibited GUS activity triggered by IAA, whereas 100 µM NPA slightly inhibited IAA-induced GUS activity. Furthermore, the suppression of GUS staining by anti-auxins and 100 µM TIBA was detected mainly in roots but not in shoots (Figure 6B, g–k).

### The BR-auxin relationship relative to *DWF4* transcription

**Figure 7:**
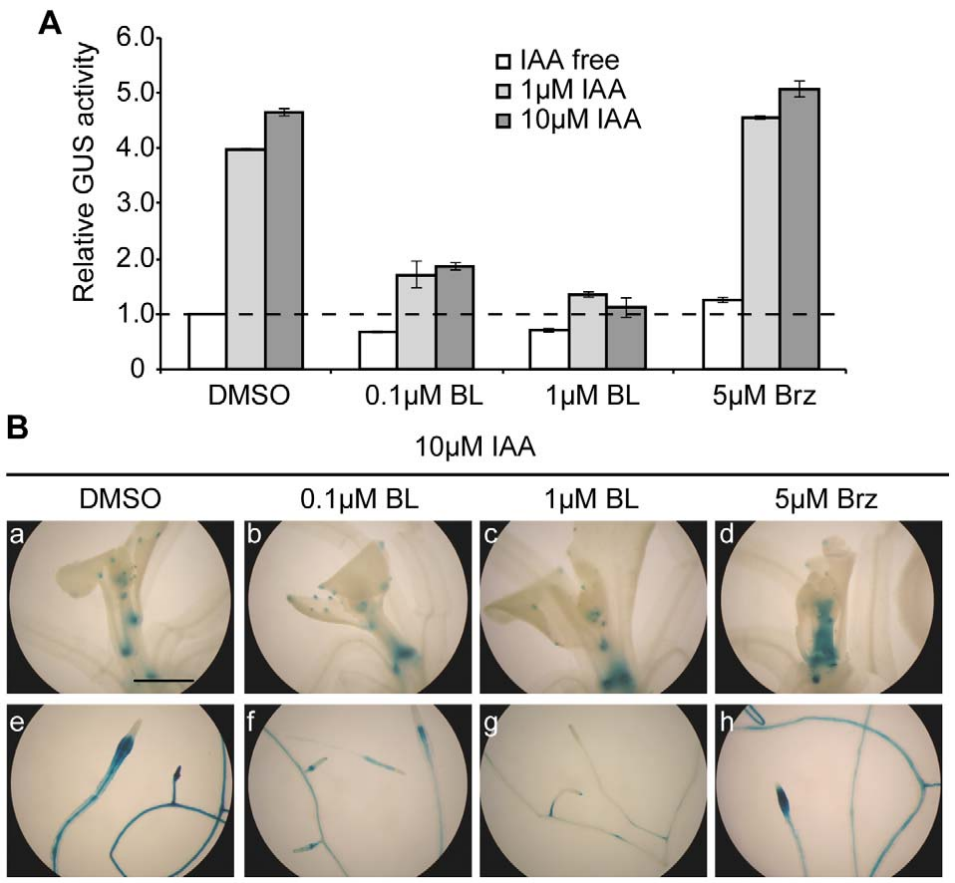
The relationship between IAA and either BL or Brz regarding DWF4::GUS activity. IAA was applied concurrently with either Brz or BL to 14-day-old *DWF4::GUS* transgenic seedlings at the indicated concentrations and then the seedlings were cultured for 1 day in liquid MS medium. GUS activity was analyzed in triplicate, biochemically (A) and histochemically (B). In the graphs, the GUS activity (the mean ± SD) of the representative result is shown as a value relative to that of the untreated control. The photographs represent the shoots (a–d) and roots (e–h) of transgenic *Arabidopsis*. The magnification is the same in all photographs, and the bar indicates 1.0 mm (a).

As described above, *DWF4* transcription was induced by auxin and Brz but suppressed by BL (Figures 3 and 4). Therefore, we applied IAA and BL simultaneously to *DWF4::GUS* seedlings for 1 day to determine which hormone is superior in regulating *DWF4* transcription. Figure 7A shows that IAA-induced GUS activity was reduced by 0.1 µM BL and was mostly suppressed by 1 µM BL. In agreement with this result, BL application attenuated IAA-induced GUS staining, particularly in roots (Figure 7B, e–g), suggesting the dominance of BR suppression over the auxin induction of *DWF4* transcription.

We then investigated the relationship between auxin and Brz effects on *DWF4* transcription. As shown in Figure 7A, Brz and IAA additively enhanced GUS activity in *DWF4::GUS* plants. Moreover, the enhancement of GUS staining by Brz was observed in the shoots but not in the roots of IAA-treated seedlings (Figure 7B, d and h), although Brz alone induced GUS activity in roots (Figure 3B). Together, these results imply that IAA and Brz up-regulate *DWF4* transcription independently.

### The roles of BRs in auxin-regulated root growth

**Figure 8:**
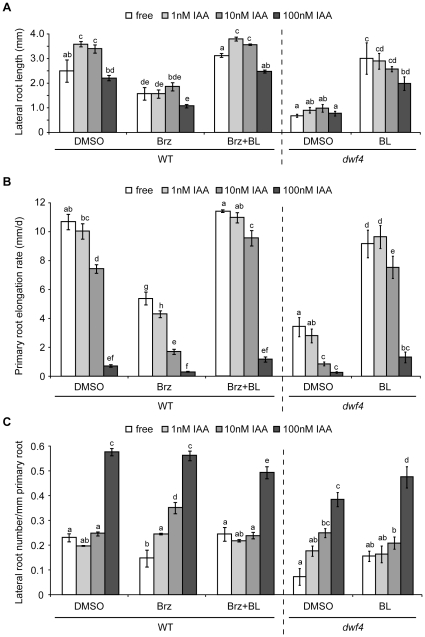
The effect of BR on auxin-regulated root elongation and lateral root formation. Seven-day-old seedlings grown on solid MS medium of wild-type (Col-0) and *dwf4* mutant *Arabidopsis* (in a Col-0 background) were transferred to the same medium containing different combinations of BL (1 nM), Brz (500 nM) or IAA (1, 10 or 100 nM) for 3 more days. The lengths of the lateral (A) and primary roots (B) and the number of lateral roots (C) of the seedlings were then measured. For the primary roots, the elongation was calculated by subtracting the root length at the initial point (day 7) from the total root length at day 10. The primary root elongation rate (B) refers to the average elongation rate (mm/d). The histograms in each graph show the means ± SE of triplicate experiments (Col-0; n = 10, *dwf4*; n = 8 to 10). Statistical analyses were performed separately for each data set obtained using wild-type and *dwf4* plants. The different letters on the tops of the histograms indicate a statistically significant difference of P<0.01 using a one-way ANOVA followed by Tukey's test.

To elucidate the physiological role(s) of IAA-induced *DWF4* transcription, we examined the root growth and morphology of wild-type (Col-0) and *dwf4* seedlings treated with different combinations of BL, Brz and IAA. As shown in Figure 8A, lateral root elongation in wild-type plants was stimulated by IAA at 1 and 10 nM, but not at 100 nM. In *dwf4* mutants, lateral root elongation was barely enhanced at any dose of IAA (Figure 8A). Consistent with the latter result, the application of 500 nM Brz to wild-type plants completely suppressed IAA-induced lateral root growth (Figure 8A). Exogenous BL suppressed the inhibitory effects of Brz and the *dwf4* mutation on lateral root elongation but affected IAA-enhanced lateral root growth differentially in the two types of seedlings: BL restored the responsiveness of IAA in Brz-treated wild-type plants but not in *dwf4* mutants (Figure 8A).

Unlike the effect on lateral roots, the growth of primary roots was inhibited dose-dependently by IAA in both wild-type and *dwf4* seedlings (Figure 8B). Administration of 500 nM Brz further enhanced IAA-induced growth inhibition in wild-type primary roots. Again, 1 nM BL rescued the suppression caused by low doses of IAA and Brz to the untreated levels, although 1 nM BL failed to rescue suppression in the presence of 100 nM IAA. BL had a similar effect on primary root elongation in *dwf4* mutants (Figure 8B).

Auxin is known to promote lateral root formation [24]. Consistently with the previous studies, we observed that 100 nM IAA significantly increased the lateral root number per unit length of primary root in both wild-type and *dwf4* plants (Figure 8C). As mentioned above, IAA enhanced *DWF4* transcription in lateral root primordia (Figure 5). Moreover, 500 nM Brz caused a slight reduction in the lateral root number in the untreated wild-type seedlings, and 1 nM BL rescued this decrease (Figure 8C). Similarly, the lateral root number was lower in *dwf4* than in wild-type plants, although this reduction was not completely recovered by application of BL (Figure 8C). However, the effects of BL, Brz and the *dwf4* mutation on lateral root formation were not observed in the presence of IAA. These results suggest that BRs and auxin cooperatively and positively regulate lateral root formation and that the BRs play a subordinate role.

### Endogenous BR content in IAA-treated *Arabidopsis* roots

As described above, IAA induced *DWF4* transcription, especially in roots. Thus, to verify whether IAA increased endogenous BR levels in roots, we measured the levels of two bioactive BRs, castasterone (CS) and BL, in IAA-treated wild-type *Arabidopsis* using liquid chromatography-electrospray ionization-tandem mass spectrometry (LC-ESI-MS/MS). In shoots, approximately 2 ng/g DW of CS was detected in two duplicate measurements, and the CS content was not affected by IAA application (Table 1). This result is consistent with the lack of induction of DWF4::GUS acitivity by IAA in shoots (Figure 5). In contrast, in the first test, CS was not detected in a root sample collected from 600 seedlings. However, a small amount of CS (<0.305 and <0.229 ng/g DW in DMSO- and IAA-treated roots, respectively) was quantified in a root sample collected from 1,200 seedlings. The CS level in the roots was approximately 10-fold lower than in the shoots (Table 1), which is consistent with data obtained using 5-week-old, soil-grown *Arabidopsis* [20]. As in the case of the shoots, the CS content in the roots was not affected by IAA application, contrary to our expectations. Another bioactive BR, BL, was not detected in any samples tested. The IAA content slightly increased in both shoots and roots when IAA was applied exogenously (Table 1).

**Table 1:** Quantification of plant hormones in wild-type *Arabidopsis* seedlings.

	Hormones	First test (600 seedlings)		Second test (1200 seedlings)	
Organs	(ng/g DW)	free	1 µM IAA	free	1 µM IAA
Shoots	CS	1.93	1.92	1.96	2.10
	BL	nd	nd	nd	nd
	IAA	200	249	96.2	101
Roots	CS	nd	nd	<0.305*	<0.229*
	BL	nd	nd	nd	nd
	IAA	291	377	264	357

*The maximum value is shown as for CS in roots (refer to Materials and Methods for detail). DW, dry weight. nd, not detected.

## Discussion

In this study, we present evidence that *DWF4* transcription is not only a part of the BR feedback control machinery but also functions in root growth directed by auxin.

We previously found that *DWF4* mRNA levels are sharply decreased by exogenous BL but are rapidly increased by Brz, indicating that *DWF4* expression plays a crucial role in BR homeostasis [9]. *DWF4* transcription has been shown to be down-regulated by a transcription factor, BZR1, which binds to the 5′ upstream sequence of *DWF4*, depending on BR levels [19]. In contrast, Brz-induced *DWF4* transcription has been reported only by Kim et al. [20], who showed that Brz enhanced both the mRNA level and histochemical staining of *DWF4::GUS*. Therefore, we investigated the kinetic behavior of *DWF4* hnRNA, which reflects its transcription rate, and the mRNA and activity levels derived from a *DWF4::GUS* transgene to elucidate the extent to which transcriptional control contributes to the feedback regulation of *DWF4* mRNA. Both *DWF4* hnRNA and *DWF4::GUS* mRNA were reduced in the same manner as *DWF4* mRNA in BL-treated *Arabidopsis* (Figures 1 and 2). In addition, GUS activity was suppressed by the same treatment (Figure 3). Together with the previous findings [19], [20], our data strongly support the idea that BL-triggered down-regulation of *DWF4* expression is largely achieved by transcriptional control.

Upon Brz treatment, similar increases were seen in the hnRNA and mRNA levels of endogenous *DWF4* (Figure 1). In addition, *GUS* mRNA and GUS activity were increased by Brz (Figures 2 and 3). These data also suggest the involvement of transcriptional control in the Brz-mediated up-regulation of *DWF4* mRNA, as previously reported by Kim et al. [20]. However, Brz increased *DWF4::GUS* mRNA to a lesser degree and later than endogenous *DWF4* mRNA (Figure 2). Weak enhancement of the *GUS* mRNA by Brz is consistent with the data from Kim et al. [20]. This result might be explained by the similar construction of the two *DWF4::GUS* transgenes used in different laboratories, which were created by fusing the 5′ upstream sequence from the start codon of *DWF4* with the *GUS* gene in the pBI101 plasmid. Although the regulatory elements for RNA polymerase II–mediated transcription exist mostly in the 5′ upstream regions of genes, the elements are occasionally found in the introns and the 3′ downstream sequence [25]–[28]. Thus, the weak induction of *GUS* mRNA implies that the Brz-induced *DWF4* transcription requires *cis*-element(s) potentially existing in its introns and/or 3′ downstream sequence in addition to those in the 5′ upstream sequence. However, we cannot exclude the possibility that post-transcriptional control is also involved in this process. As for the timing of Brz-increased *GUS* mRNA, our result conflicts with the data of Kim et al. [20]; *GUS* mRNA was increased beginning on day 2 in our case (Figure 2) but did so within 6 hours in their study. The causes of this difference are not yet known, but they might stem from the differing experimental conditions used by the two laboratories, including the length of the *DWF4* promoter used (1.7 kb in our study vs. 1.1 kb in their study) and the age of seedlings tested (14 days old vs. 7 days old). Together with previous studies, the results of our kinetic and quantitative study suggests that *DWF4* transcription functions as a part of the feedback control machinery that maintains adequate BR levels in response to both their excess and depletion, although the interpretations for some findings regarding Brz-induced *DWF4* expression remain to be determined.

To further understand DWF4 functions, we examined whether *DWF4* transcription is controlled by plant hormones other than BRs. We found that IAA alone, out of the eight plant hormones tested, induced DWF4::GUS activity (Figure 4A). In addition, GUS induction by IAA was dose- and time-dependent (Figure S1). Moreover, other bioactive auxins, including IBA, NAA and 2,4-D, promoted GUS activity, but natural auxin precursors, including Trp and IAM, hardly promoted GUS activity (Figure 4B). Together, these results suggest that both bioactive auxins and BRs regulate *DWF4* transcription. This phenomenon was further confirmed in an experiment using three anti-auxins, PCIB, PEO-IAA and BH-IAA; each of these substances repressed IAA-induced GUS activity (Figure 6). The latter two chemicals have recently been shown to directly bind to a TIR1 receptor and inhibit the function of SCF^TIR1^ ubiquitin ligase [22], [23]. Independently of our work, Chung et al. [29] recently reported that auxin induced *DWF4::GUS* expression and that the induction suppresses in several auxin signaling mutants, such as *axr6-2*. Thus, our results, together with those discussed above, strongly suggest that *DWF4* transcription is under the control of SCF^TIR1^-mediated auxin signaling [30], [31].

PAT is involved in various auxin-mediated developmental processes via the asymmetric distribution of auxin [32]. Therefore, we examined whether PAT affects *DWF4* transcription and found that two PAT inhibitors produced inconsistent results: 100 µM TIBA completely repressed the induction of DWF4::GUS activity by IAA, whereas 100 µM NPA did not (Figure 6). This discrepancy may be a reflection of the distinct functions of these inhibitors; TIBA interferes with the subcellular trafficking of auxin efflux (PIN1) and influx (AUX1) carriers, whereas NPA inhibits P-glycoproteins, such as AtPGP1 and AtPGP19, which are essential for auxin efflux [32]. Alternatively, TIBA may suppress IAA-triggered *DWF4* transcription through its secondary effects unrelated to PAT inhibition. Oono et al. [33] described that TIBA has anti-auxin activity at high concentrations, based on their finding that TIBA at 50 µM, but not at 20 µM, suppresses NAA-induced GUS activity in the root elongation zones of transgenic *Arabidopsis* that harbors the *GUS* gene driven by an auxin-inducible BA promoter. Thus, it can be speculated that *DWF4* transcription was suppressed by the anti-auxin activity of 100 µM TIBA. However, we cannot rule out other possibilities, including that the putative toxicity of TIBA at high concentrations suppressed IAA-triggered transcription of *DWF4*. Regardless, additional experiments are required to verify whether PAT is involved in this phenomenon.

BRs and auxin have been reported to interact cooperatively or antagonistically in various developmental processes [11], [13]. The physiological role of BR-auxin crosstalk through *DWF4* transcription is still unknown. Auxin-induced DWF4::GUS activity was mainly observed in roots and was most prominent in the elongation zones of both primary and lateral roots (Figure 5), consistent with the data of Chung et al. [29]. This root-specific induction was confirmed by a quantitative GUS activity measurement (Figure S2). Morphometric analysis showed that the lateral roots of wild-type seedlings were elongated by low doses (1 and 10 nM) of IAA (Figure 8A). The IAA-induced elongation of lateral roots was suppressed by either Brz treatment or a *dwf4* mutation, and this suppression was rescued by BL (Figure 8A). Moreover, PEO-IAA inhibited the IAA-stimulated induction of lateral root growth, and this inhibition was partially rescued by BL (data not shown). BRs regulate genes that encode cell wall enzymes, such as *TCH4*, *XTR6* and *XTH19*, which act during cell elongation and are expressed in roots [13], [17], [34]. Together with previous studies, our results indicate that auxin elongates lateral roots partly through enhanced BR function, which may be caused by auxin-induced *DWF4* transcription.

Most major plant hormones are reportedly involved in primary root elongation, with positive and negative effects [35]. In our experiments, exogenous IAA suppressed primary root elongation at all concentrations (Figure 8B). The IAA-induced growth retardation was further enhanced by both Brz administration and a *dwf4* mutation, and it was rescued by BL (Figure 8B). Furthermore, GUS staining in *DWF4::GUS* plants was intensified by IAA in the elongation zone of primary roots (Figure 5). These results imply that BRs also have a positive role in elongation in primary roots under the control of auxin. This idea is supported by Mouchel et al. [18], who have reported that an auxin-inducible *BRX* gene up-regulates a *CPD* gene encoding another key enzyme in BR biosynthesis and positively controls primary root elongation. While BRs are predicted to act in a positive manner, we wondered why and how exogenous IAA led to the net inhibition of primary root growth. Auxin has been reported to inhibit root growth via interactions with ethylene at different levels, including the biosynthesis, signaling and sharing of target genes [36], [37]. Jasmonic acid may also cooperate with auxin in root growth inhibition because we found that MeJA reduced DWF4::GUS activity (Figure 4A). In addition, a slight increase in JA content was observed in IAA-treated *Arabidopsis* roots (data not shown). Consistent with our results, Ren et al. [38] reported that MeJA suppresses *DWF4* expression and also inhibits primary root growth. Thus, exogenous IAA likely retards primary root growth in cooperation with endogenous hormones, such as ethylene and JA, while it concomitantly affects BRs. These results also suggest that *DWF4* transcription is one of the integration nodes in auxin-controlled primary root growth; auxin promotes *DWF4* transcription, whereas it indirectly suppresses *DWF4* expression through JA function.

As mentioned above, circumstantial evidence indicates that BRs are involved in auxin-controlled root growth (Figure 8). Therefore, we questioned whether auxin increases the bioactive BR levels in roots through the induction of BR biosynthetic genes, such as *DWF4* and *CPD*. As shown in Table 1, CS was detected in roots, but its concentration was not altered by IAA application. Although there could be several explanations for this result, it is possible that the rapid feedback machinery for BR homeostasis reduces the auxin-induced increase in CS content to the basal level. Choe et al. [39] have reported that transgenic *Arabidopsis* plants ectopically overexpressing *DWF4* (AOD4) contain lower levels of CS and BL compared with wild-type plants, although *AOD4* plants are much larger than the wild type. The expression of *BAS1*, which encodes a BR metabolic enzyme, was found to be enhanced in *AOD4* plants. These findings support the hypothesis that BR production directed by IAA was rapidly canceled via its feedback machinery. Alternatively, auxin might affect root growth through a slight increase in BR content that was not detectable in our analysis because the response of roots to plant hormones such as auxin and BRs is more sensitive than that of shoots [40], [41]. However, we cannot rule out other possibilities. For instance, Nakamura et al. [42] have demonstrated that BR intermediates participate in auxin-induced bending of the lamina joint in rice. In addition, auxin has been shown to increase the contents of several BR intermediates, such as 22-hydroxy-campesterol and 22-hydroxy-(24R)-5α-ergostan-3-one in *Arabidopsis* roots, which are the direct products of DWF4 catalytic reactions [29]. These results imply that BR intermediates are involved in the auxin-regulated root growth, although the end product of BR biosynthesis, BL rescued the inhibitory effect of Brz on the auxin-induced root elongation (Figure 8). In any case, further studies are required to answer whether and to what degree auxin may influence the contents of bioactive BRs through the regulation of its biosynthesis, and whether BR intermediates directly affect auxin-regulated root growth.

In conclusion, we kinetically verified that *DWF4* transcription functions not only in BR homeostasis but also as a site of convergence of BR-auxin crosstalk. In addition, we demonstrated that auxin-directed *DWF4* transcription is closely associated with root elongation. Although BR and auxin together influence various physiological processes occurring in roots [11], [14], [16], our study provides a novel example of their interactions. It remains to be determined, however, how and to what extent *DWF4* transcription or elevated BR levels contribute to auxin-mediated root growth; it is largely unknown which genes or proteins (enzymes) are regulated by BRs in auxin-treated roots. Transcriptome and proteome analyses of roots, in combination with genetic and pharmacological studies, should help to determine the molecular functions of BRs and the relationship between BR and auxin in root growth.

## Materials and Methods

### Chemicals

All chemicals were purchased from Wako Pure Chemical Industries, Ltd., Japan, unless specified otherwise. The plant hormones used in this study are described below. We used the following bioactive auxins and auxin precursors: IAA (Nacalai Tesque, Inc., Japan), IBA, NAA and 2,4-D (Sigma-Aldrich Co., St. Louis, MO) as bioactive auxins, and Trp (Nacalai) and IAM as auxin precursors. Other hormones used in this study included (±)-abscisic acid, 6-benzylaminopurine, BL (Brassino Co., Ltd., Japan), gibberellin A_3_ (Nacalai), MeJA, salicylic acid and 1-amino-1-cyclopropane carboxylic acid as an ethylene precursor. Deuterium-labeled CS and BL ([^2^H]_4_CS and [^2^H]_4_BL) were obtained from Dr. Hideharu Seto (RIKEN, Wako, Japan) and used as internal standards for BR quantification. The hormone inhibitors used included PCIB (Sigma), BH-IAA and PEO-IAA [22], [23] as anti-auxins, NPA and TIBA (Sigma) as PAT inhibitors, and Brz [43] as a BR biosynthesis inhibitor. To prepare stock solutions, all chemicals were dissolved in >99% DMSO, except Trp, which was dissolved in 0.1 N hydrochloric acid, and [^2^H]_4_CS and [^2^H]_4_BL, which were dissolved in 50% acetonitrile.

### Plant materials and growth conditions

The *Arabidopsis thaliana* ecotypes *Wassilewskija* (WS) and *Columbia* (Col-0) were used in this study. Seeds from a T-DNA insertion mutant of *DWF4*, *dwf4-102* (SALK_020761), were obtained from the *Arabidopsis* Biological Resource Center (ABRC; Columbus, OH). Seed sterilization and growth conditions followed the methods described by Tanaka et al. [12] for the most part, except that the final concentration of a gelling agent, gellan gum, in solid Murashige and Skoog (MS) medium was reduced from 5.0 g/L to 3.2 g/L.

### Construction of the *DWF4* promoter-driven *GUS* gene and plant transformation

A 2.5-kb DNA fragment containing the *Arabidopsis DWF4* upstream sequence was PCR amplified using high-fidelity KOD DNA polymerase (Toyobo Co. Ltd., Japan) with a pair of D4proF (5′-GCATAAAGCATAAAGGACCCGTTC-3′) and D4proR (5′-AGTTTCTCTCTCTCTCTCACTCAC-3′) primers. The PCR products were cloned into the *EcoR* V site of pBluescriptKS(+) and sequenced to confirm that the 5′ flanking region of the *DWF4* gene (GenBank accession number: AF044216) had no difference in the nucleotide level, as compared with the published sequence. A 1.7-kb DNA fragment was excised from the pBluescriptKS(+)-based plasmid using *Xba* I and *BamH* I and directionally re-cloned into the same restriction sites upstream of the promoterless *GUS* gene in the pBI101-Hm vector [44]. *Agrobacterium tumefaciens* strain EH105 was transformed with the resulting plasmid and was then subjected to vacuum infiltration [45] to generate transgenic *Arabidopsis* (WS) plants carrying the *DWF4::GUS* construct. Fifteen independent *DWF4::GUS* plants were generated, and a representative line with respect to organ-specific GUS staining was selected and used in this study.

### Hormone treatments

Fourteen-day-old *Arabidopsis* seedlings grown on MS plates were cultured with shaking at 80 rpm (Recipro Shaker NR-10; Taitec, Japan) in liquid MS medium containing hormones or inhibitors. The ratio of seedling number to the volume of liquid MS medium differed depending on the experiment: for semi-qRT-PCR analyses, the ratio was 20 plants/10 ml, for the biochemical GUS assay, the ratio was 5 plants/2.5 ml, and for the histochemical GUS assay, the ratio was 1 plant/ml. The final concentration of DMSO (used as a solvent) did not exceed 0.1%.

For morphometric analyses, *Arabidopsis* seedlings were grown under continuous light at 22°C for 7 days on vertically oriented MS plates. Seedlings were then transferred to solid MS medium containing BL, Brz and/or IAA and cultured for 3 more days under the same conditions.

For BR quantification, 10 ml of 1 µM IAA-containing liquid MS medium was applied to 20 of 14-day-old wild-type WS seedlings grown on horizontally oriented MS plates. After 1 day of incubation, the seedlings were collected and dissected at the shoot-root junction. Shoot and root samples were then freeze-dried and stored at −30°C prior to LC-ESI-MS/MS analysis.

### GUS assay

GUS (β-glucuronidase) activity was measured biochemically according to the method of Jefferson [46], except that crude protein was extracted using a buffer containing 20% methanol [47]. Histochemical GUS staining was performed following the method of Yoshimitsu et al. [48], except that a substrate solution containing 0.5 mM each of potassium ferricyanide and potassium ferrocyanide was used.

### Semi-qRT-PCR

RNA extraction and semi-qRT-PCR were performed according to Tanaka et al. [9]. RNA samples were incubated with RNase-free DNase I (Takara Bio Inc., Japan) and were used as a direct template for PCR to confirm the complete removal of contaminating genomic DNA. The DNA-free RNA samples were then applied to semi-qRT-PCR. The sequences of the primers, number of cycles and annealing temperature for semi-qRT-PCR for each gene are listed in Table S1.

### Morphometric analysis

Photographs of hormone-treated seedlings were taken with a digital camera (PowerShot S5 IS, Canon) and used to measure the numbers and lengths of lateral roots and the lengths of primary roots using ImageJ software (NIH, Maryland, USA). Statistical analysis of a one-way ANOVA followed by Tukey's test was carried out using PASW statistics 18.0 software.

### Quantification of BR content

Upon beginning extractions, approximately 5 ng/g DW of [^2^H]_4_CS and 1 ng/g DW of [^2^H]_4_BL were added as internal standards. Neutral extracts containing bioactive BRs were prepared by sequential extraction with hydrophobic, cation-exchange and anion-exchange cartridges, as previously described [49]. The extract was dried, resuspended in 1 ml of CHCl_3_ and loaded onto a normal-phase extraction cartridge (SepPak Silica, Waters, Milford, MA, USA). BRs were eluted with 2 ml of CHCl_3_∶MeOH = 9∶1 (v/v) after washing with 1 ml of CHCl_3_. The eluate was dried and dissolved in 100 µl of 50% methanol and subjected to HPLC equipped with an ODS column (CAPCELL PAK C18, 4.6×250 mm, Shiseido, Tokyo, Japan). Separation was performed using a gradient of increasing acetonitrile to water with a flow rate of 1 ml/min. Ten percent acetonitrile was eluted for 10 min, and its concentration was increased to 40% for 1 min. Next, the concentration of acetonitrile was increased to 60% for 20 min. After washes with 90% acetonitrile for 10 min, the initial concentration (10%) of acetonitrile was restored and allowed to equilibrate for 10 min. The eluate was collected at a rate of 1 tube/min. After drying the solvent, the 23^rd^ to 30^th^ fractions were analyzed by LC-ESI-MS/MS (Agilent, 1200–6410), as shown in Tables S2 and S3. Ratios were calculated by comparing the peak areas for endogenous CS and BL to their respective internal standards. The concentration of endogenous CS in shoots was evaluated using the average of three specific product ions, while that in roots was determined using the strongest ion (463/129) among the three as a result of low peak area. Thus, the CS amount in roots is displayed as the maximum value in Table 1.

## Supporting Information

Figure S1 Dose- and time-dependent effects of IAA on DWF4::GUS activity. The *DWF4::GUS* transgenic plants (14 days old) were incubated in liquid MS medium containing various concentrations of IAA for the indicated periods. Then, GUS activity was measured biochemically. The GUS activity is shown as a value relative to the activity of untreated plants (A) and at the initial period (B).
(TIF)

Figure S2 The DWF4::GUS activity in shoots and roots of IAA-treated seedlings. Fourteen-day-old *DWF4::GUS* transgenic plants were incubated in liquid MS medium containing IAA. After 1 day of incubation, the seedlings were dissected at the shoot-root junction. GUS activities in the separated shoots and roots were measured biochemically. The GUS activity is shown as a value relative to the activity of each untreated control.
(TIF)

Table S1 Primer sequences, cycle numbers and annealing temperatures used in the semi-qRT-PCR analyses.
(TIF)

Table S2 LC conditions using LC-ESI-MS/MS.
(TIF)

Table S3 MS conditions using LC-ESI-MS/MS.
(TIF)
