# Supplementary figures and images for: Transcription of DWARF4 Plays a Crucial Role in Auxin-Regulated Root Elongation in Addition to Brassinosteroid Homeostasis in Arabidopsis thaliana
Source: PLoS One. 2011 Aug 31;6(8):e23851. doi: 10.1371/journal.pone.0023851 (PMC3166115; doi:10.1371/journal.pone.0023851)

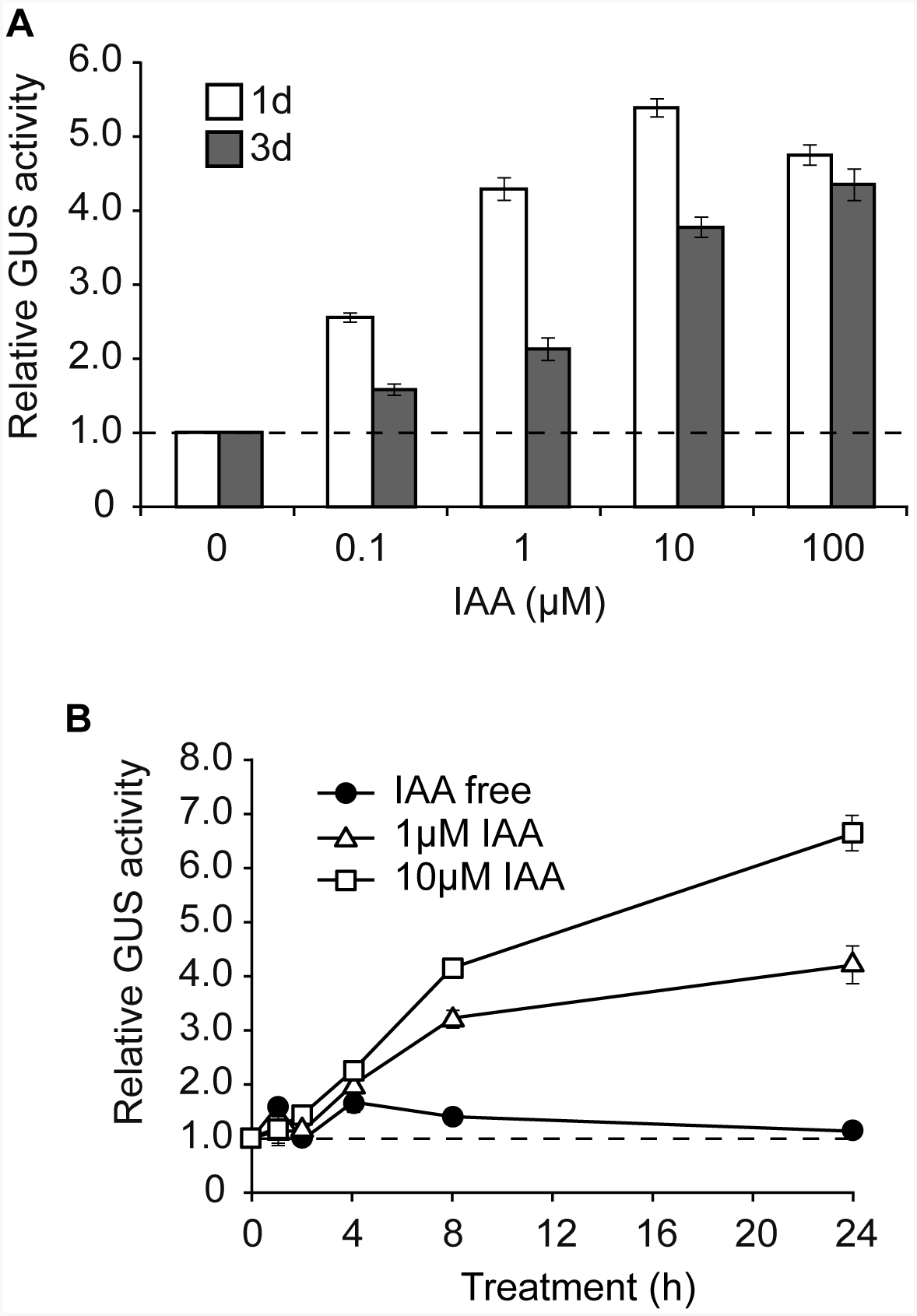

Supplement: Figure S1 — Dose- and time-dependent effects of IAA on DWF4::GUS activity. The DWF4::GUS transgenic plants (14 days old) were incubated in liquid MS medium containing various concentrations of IAA for the indicated periods. Then, GUS activity was measured biochemically. The GUS activity is shown as a value relative to the activity of untreated plants (A) and at the initial period (B). (TIF) [file pone.0023851.s001.tif]

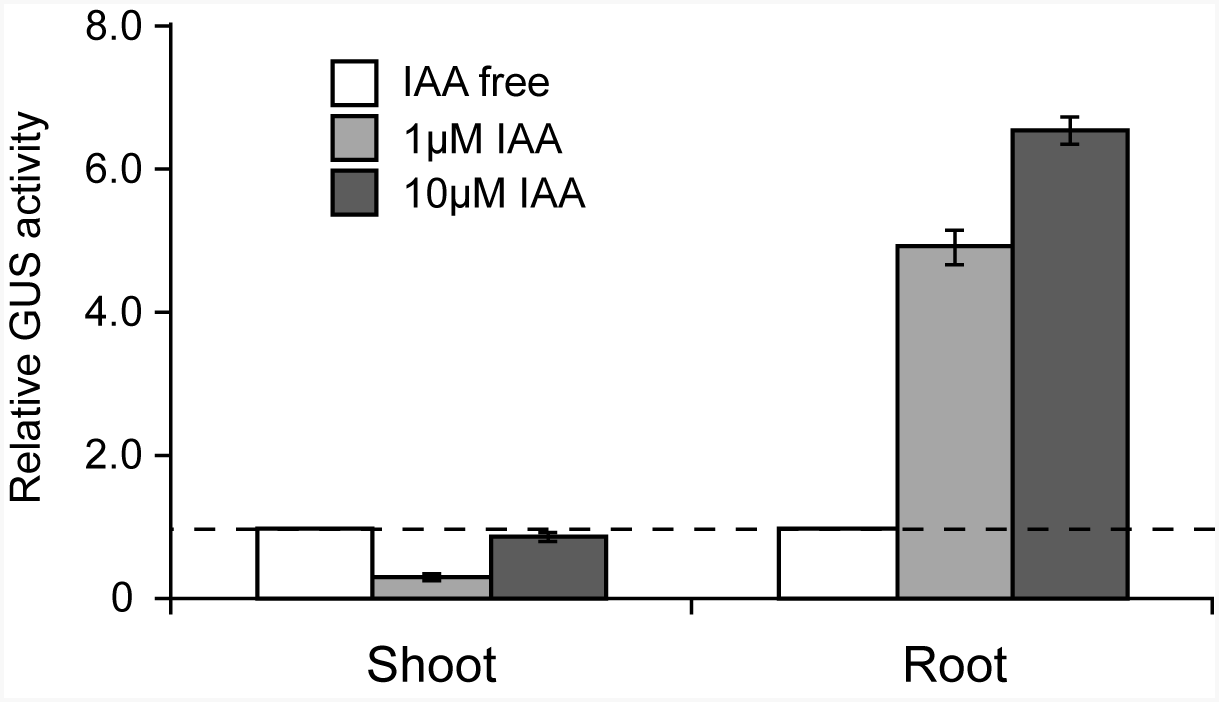

Supplement: Figure S2 — The DWF4::GUS activity in shoots and roots of IAA-treated seedlings. Fourteen-day-old DWF4::GUS transgenic plants were incubated in liquid MS medium containing IAA. After 1 day of incubation, the seedlings were dissected at the shoot-root junction. GUS activities in the separated shoots and roots were measured biochemically. The GUS activity is shown as a value relative to the activity of each untreated control. (TIF) [file pone.0023851.s002.tif]

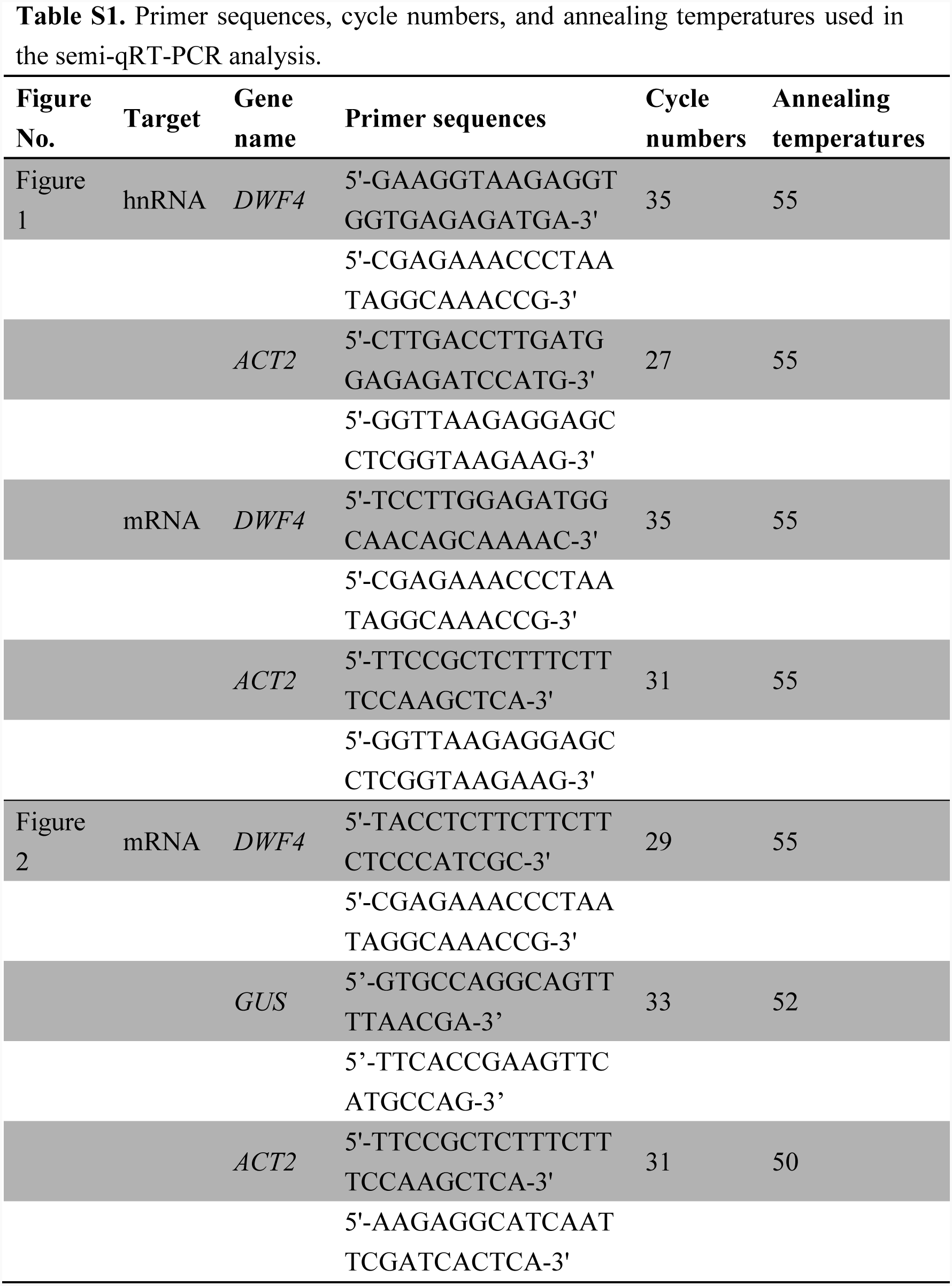

Supplement: Table S1 — Primer sequences, cycle numbers and annealing temperatures used in the semi-qRT-PCR analyses. (TIF) [file pone.0023851.s003.tif]

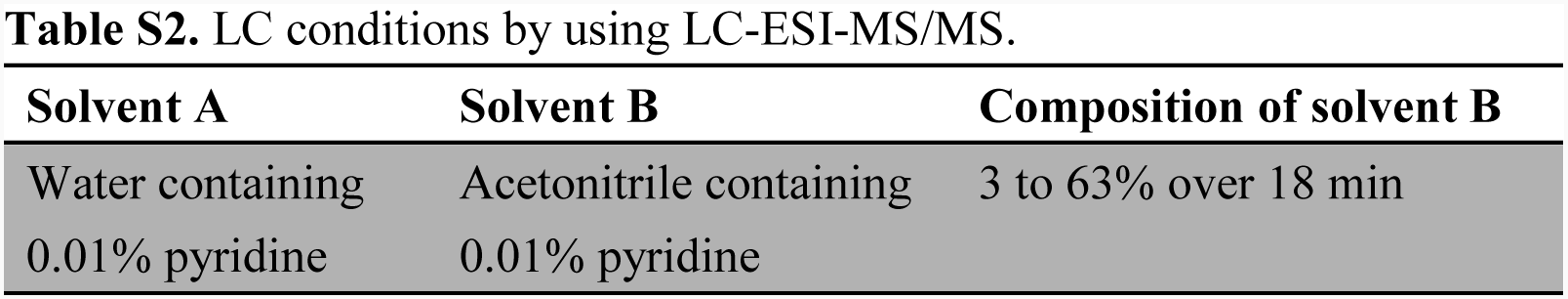

Supplement: Table S2 — LC conditions using LC-ESI-MS/MS. (TIF) [file pone.0023851.s004.tif]

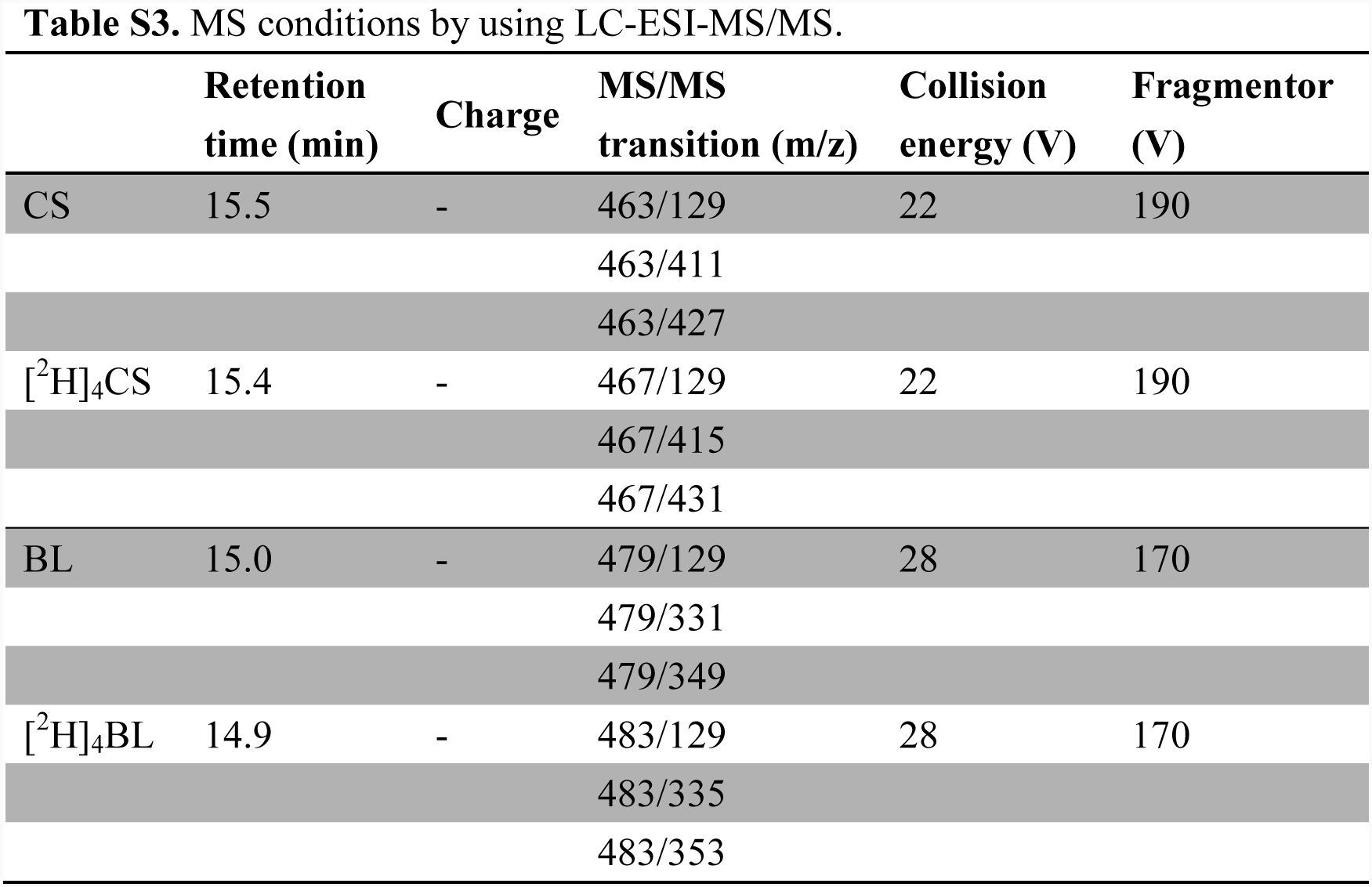

Supplement: Table S3 — MS conditions using LC-ESI-MS/MS. (TIF) [file pone.0023851.s005.tif]
